# Travel related histoplasmosis – a diagnostic challenge in a patient with tumor necrosis factor alpha (TNF-α) inhibitor therapy

**DOI:** 10.1186/s40794-022-00178-2

**Published:** 2022-09-15

**Authors:** Eveline Hofmann, Konrad Mühlethaler, Matthias Pollak, Daniel Ott, Nora Bienz, Stefan Zimmerli, Cédric Hirzel

**Affiliations:** 1 Department of Infectious Diseases, Inselspital, Bern University Hospital, University of Bern, Bern, Switzerland; 2 Department of Clinical Microbiology, Institute for Infectious Diseases, University of Bern, Bern, Switzerland; 3 Department of Hematology and Central Hematology Laboratory, Inselspital, Bern University Hospital, University of Bern, Bern, Switzerland; 4 Department of Diagnostic, Interventional and Pediatric Radiology, Inselspital, Bern University Hospital, University of Bern, Bern, Switzerland; 5 Department of Intensive Care Medicine, Inselspital, Bern University Hospital, University of Bern, Bern, Switzerland

**Keywords:** Histoplasmosis, Immunocompromised, TNF-α inhibitor, Returning traveler, Hemophagocytic lymphohistiocytosis

## Abstract

**Introduction:**

In a non-endemic setting, disseminated histoplasmosis is a rare travel-related health problem of immunosuppressed returnees from endemic regions.

**Methods:**

We describe the case of a 68-year-old man with rheumatoid arthritis and tumor necrosis factor alpha (TNF-α) inhibitor treatment-related immunodeficiency, who suffered from disseminated histoplasmosis after traveling to Brazil. Based on this case, we discuss challenges and pitfalls associated with the diagnosis of disseminated histoplasmosis in a non-endemic setting.

**Results:**

The disease mimicked a hemophagocytic lymphohistiocytosis (HLH) like syndrome. *Histoplasma capsulatum* was microscopically detected in bronchoalveolar fluid and bone marrow aspirate smears, but was initially misclassified as *Leishmania* spp., another class of pathogens, which may cause HLH like syndromes in immunocompromised individuals.

**Discussion:**

Since the clinical symptoms of histoplasmosis are nonspecific and physicians in non-endemic regions might not be familiar with this disease pattern, there is a risk of delayed diagnosis of travel related cases. Taking a thorough travel history is key in unclear cases of illness in immunocompromised patients.

## Background

In patients who receive treatment with tumor necrosis factor alpha (TNF-α) inhibitors, disseminated histoplasmosis is a rare but life-threatening complication. TNF-α and interferon-gamma (INF-γ) play a critical role in defense against *Histoplasma capsulatum* [1]. TNF-α blockade hampers the activation and function of macrophages, an essential step in the development of an efficient immune response [2]. In 2008, a cluster of histoplasmosis cases among patients receiving TNF-α inhibitors led the United States Food and Drug Administration (FDA) to issue a Boxed Warning. However, the geographic distribution is variable, and especially in non-endemic regions, many physicians may not be aware of this risk. As a result, there could be a delay in diagnosis of these cases. Patients from non-endemic countries may lack awareness regarding the risk of exposure and infection when travelling internationally.

## Case report

A 68-year-old man was admitted to a regional hospital in Switzerland after falling at home. Four days earlier, his primary care physician suspected an infection-exacerbated chronic obstructive pulmonary disease (COPD) and prescribed an antibiotic therapy (moxifloxacin 400 mg/day). Besides the COPD, the patient was known for a rheumatoid arthritis, which was treated with methotrexate (20 mg/week) and infliximab (TNF-α inhibitor; 300 mg every ten weeks). Furthermore, the patient had diabetes, an obstructive sleep apnea syndrome, and a coronary heart disease.

At admission, the patient was febrile, had hypotension and tachycardia. Laboratory analysis revealed thrombocytopenia (49 × 10^9^/L), a white blood cell count within the normal range (4.4 × 10 9/L), and a hemoglobin concentration of 13.8 g/dL. There was a mild coagulopathy (international normalized ratio, INR; 1.3), electrolyte disturbances (sodium 126 mmol/L, calcium 2.1 mmol/L), decreased renal function (serum creatinine 122 µmol/L, eGFR 50 mL/min), elevated bilirubin (25.9 µmol/L), elevated liver enzymes (aspartate aminotransferase 198 U/L, alanine aminotransferase 225 U/L), and inflammation parameters (C-reactive protein, 87 mg/L). A CT scan showed bronchial wall thickening and mild splenomegaly. In the absence of a clear focus of infection, a COPD exacerbation was suspected and moxifloxacin was changed to ceftriaxone (2 g/day) in combination with prednisolone (40 mg/day), and methotrexate was discontinued. However, there was clinical deterioration over the next days with spiking fever and progressive somnolence. The patient developed anemia (hemoglobin 10.1 g/dL), disseminated intravascular coagulation (D-dimer 75′140 ng/mL, INR 1.6, fibrinogen 1.7 g/L), massive hyperferritinemia (296′130 µg/L), and hyperbilirubinemia (96 µmol/L).

These findings were interpreted as a hemophagocytic lymphohistiocytosis (HLH) of unknown cause. The antibiotic therapy was empirically escalated to piperacillin/tazobactam (3 × 4.5 g q8h) and doxycycline (2 × 100 mg q12h), and high dose dexamethasone treatment (20 mg/day) was initiated. Furthermore, a bone marrow biopsy was sent for analysis to our center (Bern University Hospital). Multiple microbiological tests were carried out (including blood and urine cultures, pneumococcal and legionella urinary antigens, serologies for hepatitis A, B, C, and E, HIV, syphilis, *Bartonella henseale* and *quintana*, brucellosis, *Coxiella burnetii*, *Leishmania* spp., human cytomegalovirus, and Epstein-Barr virus), without finding any evidence for an infection.

**Fig. 1:**
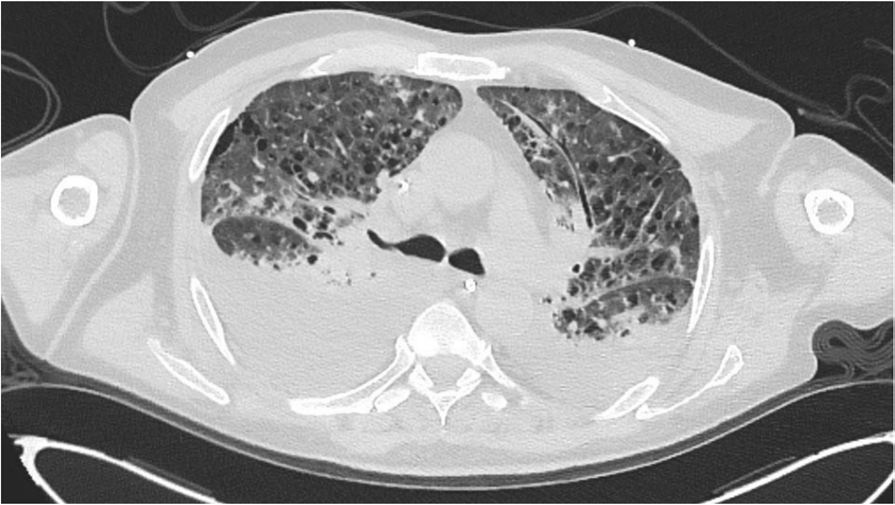
CT scan at hospital admission. Chest CT scan shows extensive bilateral pulmonary infiltrates with large pulmonary effusions and mediastinal lymphadenopathy

**Fig. 2:**
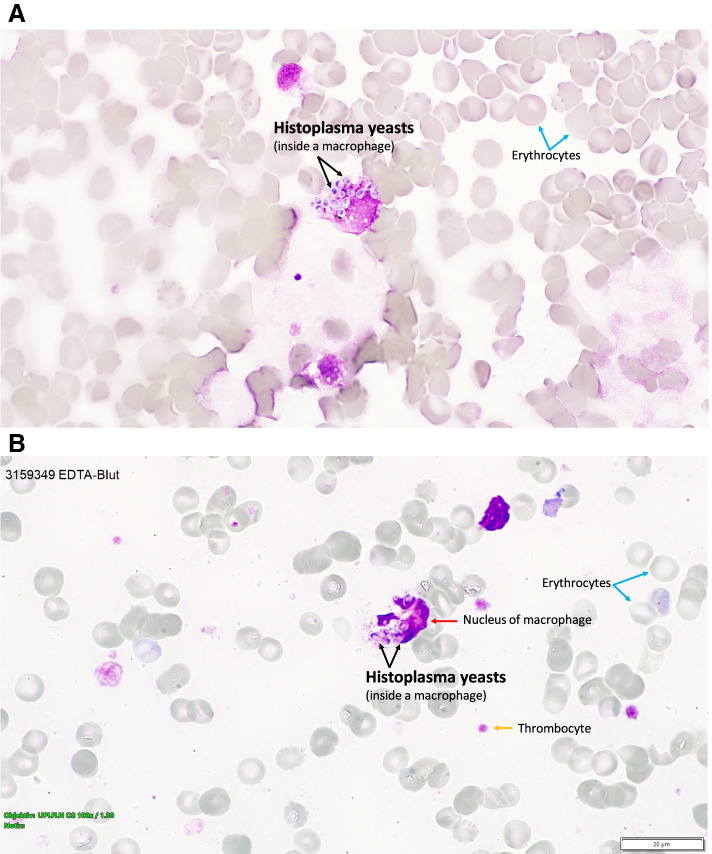
Morphology of Histoplasma capsulatum yeasts. **A** Hemorrhagic (erythrocytes blue arrows) bronchoalveolar fluid with intracellular *Histoplasma capsulatum* (black arrow) yeasts measuring 3-4 µm. **B** Peripheral blood smear with *Histoplasma capsulatum* yeasts (black arrows) within a macrophage (red arrow), eryhthrocytes (blue arrows), and thrombocytes (yellow arrows). The *Histoplasma capsulatum* yeasts exhibit the typical ovoid shape

Due to respiratory failure nine days after admission with need for mechanical ventilation, the patient was referred to our tertiary care hospital (Bern University Hospital). A repeated CT scan showed extensive bilateral pulmonary infiltrates with large pulmonary effusions and mediastinal lymphadenopathy (Fig. 1). A blood smear revealed intracellular microorganisms (3–4 µm in diameter) within phagocytes. Similar pathogens were also visible in smears of bronchoalveolar fluid and in bone marrow aspirate (Fig. 2A). Tuberculosis diagnostics (PCR, smears, and cultures) from bronchoalveolar fluid were negative. Based on the morphology of these microorganisms, a *Leishmania* spp. infection was suspected. Since leishmaniasis is not endemic in Switzerland, we reached out to the patients’ family for additional information on his travel history. It turned out that the patient travelled alone and used to spend wintertime in Brazil (Bahia) until one month before admission (November 2019). Since the patient was in critical condition and preliminary laboratory reports supported the diagnosis of a hemophagocytic lymphohistiocytosis triggered by visceral leishmaniasis, we initiated a treatment with liposomal amphothericin B (5 mg/kg/day). However, the patient showed progressive clinical deterioration with acute respiratory distress syndrome and multi-organ failure. In order to better characterize the microscopically visible microorganisms, a microbiologist with expertise in parasitology and mycology was consulted and microscopically identified the pathogens as intracellular *Histoplasma capsulatum* yeasts. This diagnosis was confirmed by a positive histoplasma antigen test in urine (5.7 EIA/U, cutoff > 2EIA/L) and in bronchoalveolar fluid (4.2 EIA/U), and by fungal cultures of blood and pleural effusion. PCR for *Leishmania* spp. in blood was negative.

Unfortunately, the patient’s condition worsened rapidly and he died seven days after the onset of respiratory failure requiring intubation and ventilation, and six days after starting treatment with liposomal amphotericin B, which is the treatment of choice for severe histoplasmosis (and visceral leishmaniasis).

## Discussion

*Histoplasma capsulatum, *The fungus that causes histoplasmosis, is ubiquitous, but it’s most commonly encountered in specific endemic areas, including North-, Central- and South America, Africa, India and Southeast Asia [3]. In Europe, there are only a few reports of autochthonous histoplasmosis cases [4]. *H. capsulatum*, a dimorphic fungus, proliferates best in soil contaminated with bird or bat droppings [5]. At lower temperatures, *H. capsulatum* presents as mold and generates conidia. The lungs are the port of entry for *H. capsulatum* by inhalation of conidiae in the vast majority of cases. Only a minority (< 5%) of exposed individuals develop symptomatic infection [6]. There is a wide spectrum of clinical diseases, ranging from asymptomatic self-limited illnesses to life-threatening infections dependent on the intensity of exposure and the host’s immune response. Following heavy exposure of non-immune individuals, subjects usually develop respiratory symptoms and diffuse pulmonary involvement within two weeks. In severe cases, the lung disease may progress and respiratory failure or extra pulmonary dissemination may occur. After low-level exposure, pulmonary disease is more commonly mild or asymptomatic. In these cases, radiographs show single or few areas of patchy opacities which subsequently evolve into nodules. Hilar and mediastinal lymphadenopathy may be present in mild and severe cases. Patients with underlying emphysema may experience progressive pulmonary disease (chronic pulmonary histoplasmosis) presenting with chronic cough, dyspnea, fever, and fatigue. This clinical manifestation mainly affects the upper lobes and resembles reactivation of tuberculosis with formation of cavitation [6]. Other manifestations of histoplasmosis include pericarditis, mediastinal histoplasmosis (mediastinal adenitis, mediastinal granuloma, or fibrosing mediastinitis), histoplasmosis of the central nervous system, or rheumatologic manifestation such as arthritis [3, 6].

Immunocompromised individuals with untreated pulmonary histoplasmosis, usually develop progressive disseminated disease. Signs of dissemination may include hepatosplenomegaly, extra pulmonary lymphadenopathy, skin lesions and bone marrow suppression [6]. Diffuse pulmonary opacities in the absence of high-inoculum exposure may suggest the presence of disseminated disease [6]. TNF-α blockade results in a compromised activation of macrophages, which is essential for eliciting effective cell-mediated immunity [2]. Interestingly, the risk of dissemination depends on the type of TNF-α inhibitor, with an increased risk with anti-TNF-α monoclonal antibodies (infliximab and adalimumab) compared to soluble TNF-α receptors (etanercept) [7]. Vergidis et al. retrospectively analyzed 98 cases of histoplasmosis in patients receiving TNF-α inhibitors. Most patients had underlying rheumatoid arthritis and more than 75% had disseminated disease [8].

Since the clinical symptoms of histoplasmosis are nonspecific, there is a risk of delayed diagnosis of travel-related cases in non-endemic regions and taking a detailed travel history is crucial. A prolonged febrile illness or pneumonia in patients treated with TNF-α inhibitors should trigger physicians to consider *Histoplasma capsulatum* infection as a possible cause of illness, especially when the patient had traveled to endemic regions [7].

**Fig. 3:**
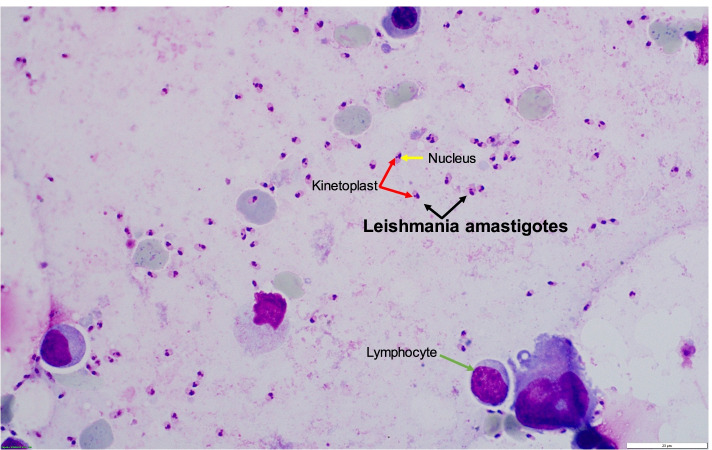
Leishmania amastigotes in a bone marrow aspirate. In the individual amastigotes (black arrows), the characteristic kinetoplast (red arrows) and the nucleus (yellow arrows) are clearly visible

Our case illustrates that pathogen assignment by light microscopy might be difficult at first glance, even when *Histoplasma capsulatum* is visible in blood smears. This might be especially true in settings where this pathogen is rarely encountered. The initial microscopic misidentification of *Histoplasma capsulatum* yeasts as *Leishmania* spp. might have been due the fact that both pathogens can predominantly be found within macrophages and that both pathogens can be encountered in Brazil [9, 10]. However, there are important differences in morphology of these microorganisms, which enable reliable differentiation between *H. capuslatum* and *Leishmania* spp. (Fig. 3): Leishmania amastigotes are slightly smaller (2–3 µm) than the *H. capsulatum* var. *capsulatum* yeasts (2–4 µm) and lack the ovoid caspule-like structure which is characteristic for *Histoplasma* spp. [10, 11]. Furthermore, the large nucleus and prominent deeply stained rod-like organelle called kinetoplast, typical of Leishmania amastigotes, is not present in *H. capsulatum* yeast cells [11]. We would like to highlight that the presence of a kinetoplast is an important criterion for microscopic identification of *Leishmania* spp.. The microscopic differential diagnoses for suspected *H. capsulatum* include *Candida glabrata*, *Cryptococcus* spp*.*, *Pneumocystis jirovecii*, *Coccidioides* spp., *Blastomyces dermatitis*, *Talaromyces marneffei*, and *Toxoplasma gondii *[10, 12]. Antigen detection has a sensitivity of 80% and a specificity of > 90% in average, and may be useful for rapid diagnosis of histoplasmosis. However, test performance varies widely depending on the product used, the analyzed specimen, the presence of immunosuppression such as AIDS, and disease severity [13].

The clinical symptoms and laboratory findings of our patient were compatible with the diagnosis of hemophagocytic lymphohistiocytosis (HLH) syndrome. Both, histoplasmosis and leishmaniasis can mimic an HLH like syndrome [9, 14]. HLH is characterized by excessive inflammation and tissue destruction due to abnormal immune activation. The dysregulated immune state is caused by insufficient downregulation of activated macrophages and lymphocytes [15]. The diagnosis of a HLH syndrome is based on the presence of typical clinical findings in the setting of elevated inflammatory markers as used in the HLH-2004 trial [16]. Important diagnostic criteria include fever, peripheral blood cytopenia, hemophagocytosis in bone marrow, hyperferritinemia, and increased soluble CD25. The HLH syndrome includes conditions which respond to HLH-directed immunosuppressive therapy (defined as “HLH disease”), but also conditions which require entirely different treatment modalities (e.g. histoplasmosis), and which are subsumed under the term of “HLH disease mimics” [17]. Histoplasmosis associated HLH is a rare disorder with high mortality (⁓30%) and has mainly been described in patients with the acquired immunodeficiency syndrome (AIDS) [14]. For treatment of severe histoplasmosis, liposomal amphotericin B (3.0 mg/kg daily) is recommended for 1–2 weeks, followed by oral itraconazole [3]. Itraconazole should be continued for at least 12 months and until clinical symptoms have resolved, antigenemia has cleared and antigenuria has decreased to < 4 ng/mL [3]. The benefit of adjunct immunosuppressive therapy or administration of intravenous immunoglobulin (IVIG) in patients with histoplasmosis triggered HLH remains unclear [14].

Before starting treatment with TNF-α inhibitors, patients should be evaluated for previous exposure to *H. capsulatum*. This includes a thorough travel history for patients living in non-endemic regions [7]. Since *H. capsulatum* proliferates best in soil contaminated with bird or bat droppings, activities that have been associated with exposure include exploring caves, excavation, construction, demolition, remodeling, wood cutting and gathering, and cleaning structures that are encrusted with bird or bat guano [5]. If there is evidence for possible exposure or previous illness, a chest x-ray should be performed and assessed for possible signs of latent histoplasmosis, such as calcified nodules or lymph nodes, before starting therapy [7]. If a patient has been diagnosed with histoplasmosis during the 2 years preceding TNF-α blocker therapy, or if the clinical, radiographic, or laboratory findings suggest the patient may have had histoplasmosis during that interval, present guidelines suggest to consider antifungal prophylaxis [3]. In people living with HIV and CD4 cell counts < 150 cells/mm3 prophylaxis with itraconazole is recommended in specific areas of endemicity where the incidence of histoplasmosis is 110 cases per 100 patient-years [3].

In summary, we highlight that the correct and timely diagnosis of histoplasmosis can be challenging in non-endemic settings, especially when the infection presents with a rare manifestation such as HLH. In these cases, taking a thorough travel history is key. Both pathogens, *H. capsulatum* and *Leishmania* spp., share overlapping endemic areas and may trigger HLH. Correct pathogen identification by light microscopy can be challenging, but the presence of specific pathogen characteristics such as the capsule-like structure in *H. capsulatum* or the kinetoplast in Leishmania amastigotes are helpful to differentiate these pathogens. Consulting an experienced microbiologist with expertise in this area is highly recommended and may help to accelerate the diagnostic process.

## Data Availability

Data is available from the corresponding author on reasonable request.

## Abbreviations

AIDS: Acquired immunodeficiency syndrome
COPD: Chronic obstructive pulmonary disease
HIV: Human Immunodeficiency Virus
IVIG: Intravenous immunoglobulin
FDA: Food and Drug Administration
HLH: Hemophagocytic lymphohistiocytosis
TNF-α: Tumor necrosis factor alpha
